# Supplementary material for: Mitochondrial haplotype and mito-nuclear matching drive somatic mutation and selection throughout aging
Source: bioRxiv. 2023 Mar 8:2023.03.06.531392. Preprint. [Version 2] doi: 10.1101/2023.03.06.531392 (PMC10028854; doi:10.1101/2023.03.06.531392)
Supplement: Supplementary file 1 [file NIHPP2023.03.06.531392v2-supplement-1.pdf]

bioRxiv preprint doi: https://doi.org/10.1101/2023.03.06.531392; this version posted March 8, 2023. The copyright holder for this preprint (which was not certified by peer review) is the author/funder, who has granted bioRxiv a license to display the preprint in perpetuity. It is made available under aCC-BY 4.0 International license.

Supplementary Figures

Fig S1

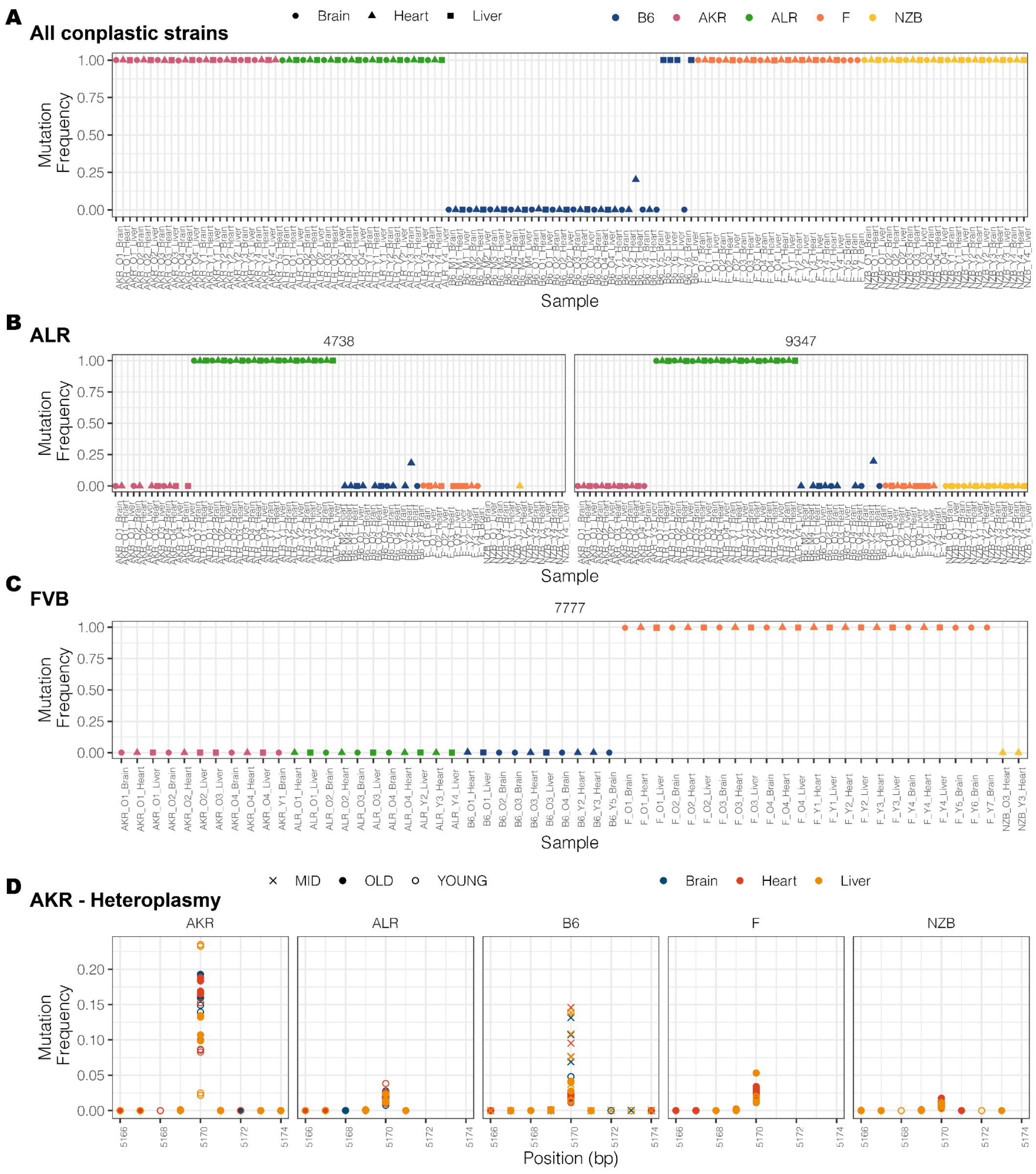

Fig S1: Validation of conplastic strains. The mutation frequency, calculated as the number of alternative alleles divided by the number of duplex mt-genomes at the position, was checked for each haplotype site. (A) The mutation frequency for the T>C mutation at position 9460 in the gene *mt-ND3.* All conplastic strains differ from B6 (blue) at this position. B6 young liver samples that had this haplotype present were excluded from the study due to potential contamination. (B) The mutation frequency for ALR (green) haplotype sites positions 4738 (*mt-ND2*) and 9347 (*mt-COXIII)*. The B6 young heart sample with presence of this haplotype was excluded from the study due to potential contamination. (C) The mutation frequency for the FVB (orange) haplotype site at position 7777 (*mt-Atp8)*. (D) Comparison of the heteroplasmic allele frequency in the *OriL*. As expected, AKR has the highest heteroplasmic allele frequency compared to other strains including B6.

bioRxiv preprint doi: https://doi.org/10.1101/2023.03.06.531392; this version posted March 8, 2023. The copyright holder for this preprint (which was not certified by peer review) is the author/funder, who has granted bioRxiv a license to display the preprint in perpetuity. It is made available under aCC-BY 4.0 International license.

Fig S2

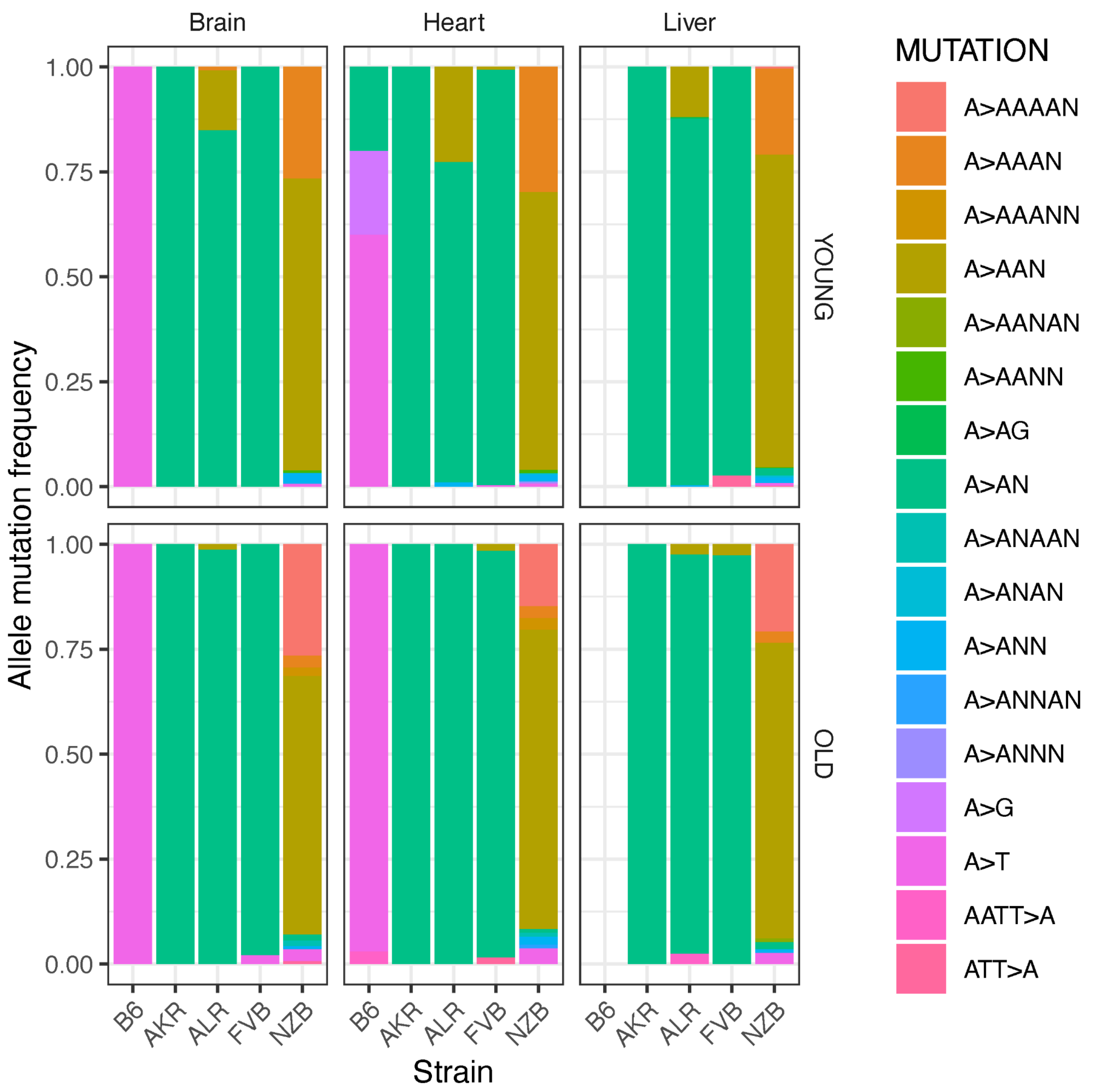

Fig S2: Proportion of alleles in the tRNA^{Arg} 8 base mutation peak region. The proportion of each allele present in the A repeat region of the DHU arm of *mt-tRNA^{Arg}* was calculated as the total number of each mutation type divided by the total number of mutations that occur in this region. This proportion was calculated for each experimental condition. Mutations that occur at a frequency > $1x10^{-3}$ were excluded from the analysis, including the previously known heteroplasmic frequency in *mt-tRNA^{Arg}* across conplastic strains[35]. The mutation key (REF>ALT) denotes the mutation that occurs in *mt-tRNA^{Arg}*, Ns in the ALT allele represent bases where a duplex consensus could not be reached.

bioRxiv preprint doi: https://doi.org/10.1101/2023.03.06.531392; this version posted March 8, 2023. The copyright holder for this preprint (which was not certified by peer review) is the author/funder, who has granted bioRxiv a license to display the preprint in perpetuity. It is made available under aCC-BY 4.0 International license.

Fig S3

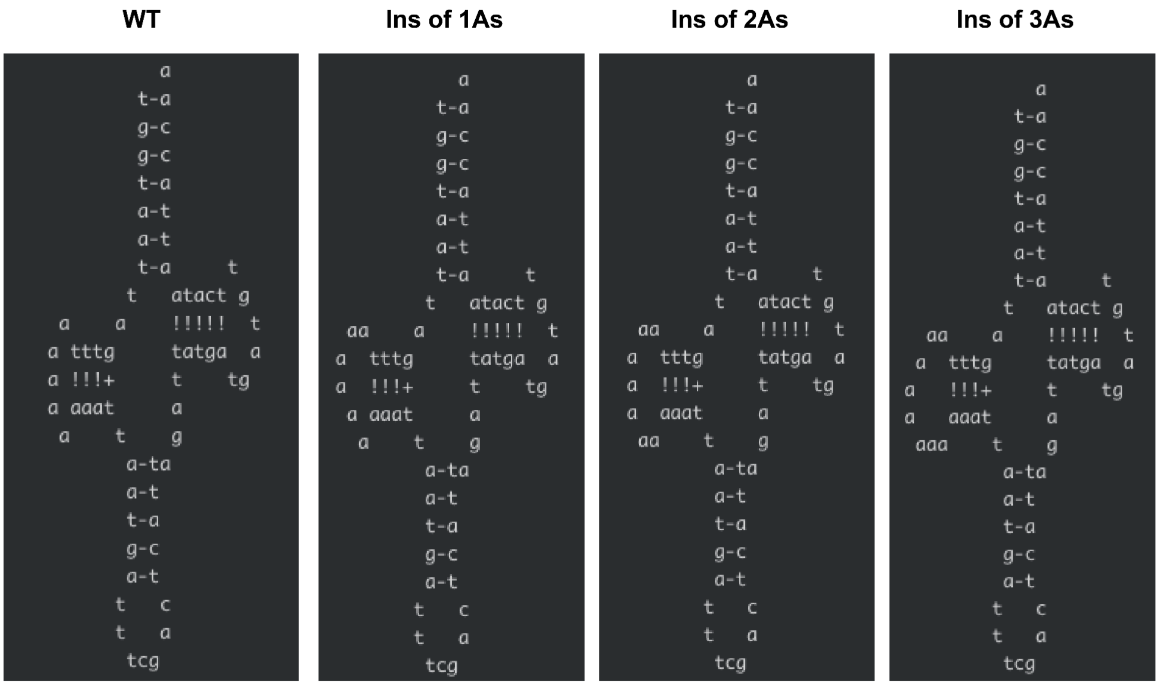

Fig S3: Computationally predicted changes in $tRNA^{Arg}$ structures. Structures were created using ARWEN (v1.2) [54]. Structures were created for the 3 most abundant mutation types across conditions (Fig S2): 1-3 A insertion in the DHU A-repeat region. As were inserted at position 9819 since we lack the resolution to locate the exact position an A-insertion occurs.

bioRxiv preprint doi: https://doi.org/10.1101/2023.03.06.531392; this version posted March 8, 2023. The copyright holder for this preprint (which was not certified by peer review) is the author/funder, who has granted bioRxiv a license to display the preprint in perpetuity. It is made available under aCC-BY 4.0 International license.

Fig S4

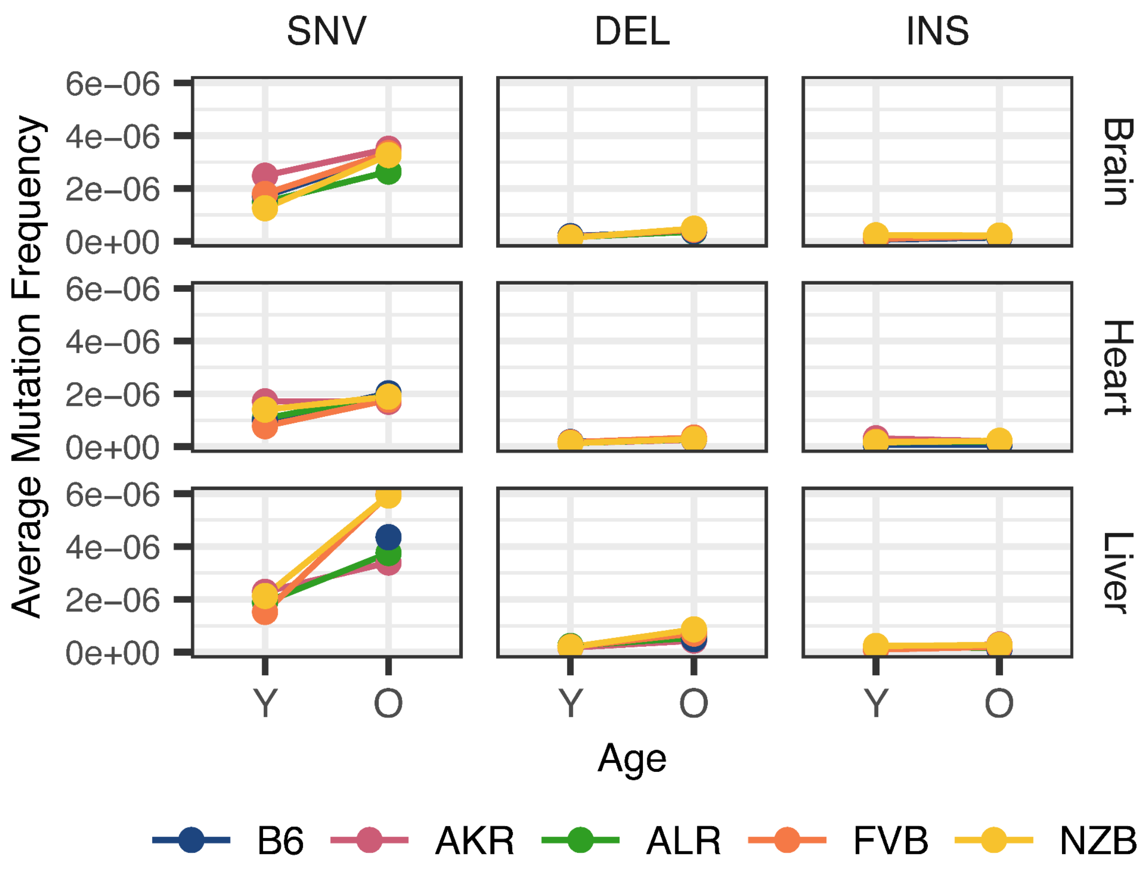

Fig S4: Change in SNVs, deletions, and insertions with age across all tissues. The average mutation frequency is compared between mt-haplotypes for different classes of mutations: single nucleotide variants (SNVs), deletions, and insertions. The average mutation frequency was calculated as the total mutation count in a class divided by the total duplex bp depth.

bioRxiv preprint doi: https://doi.org/10.1101/2023.03.06.531392; this version posted March 8, 2023. The copyright holder for this preprint (which was not certified by peer review) is the author/funder, who has granted bioRxiv a license to display the preprint in perpetuity. It is made available under aCC-BY 4.0 International license.

Fig S5

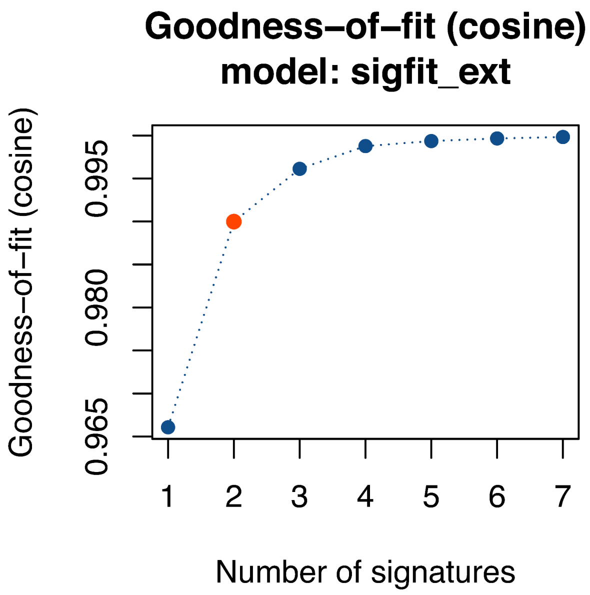

Fig S5: Estimation of the optimal number of mutational signatures. Observed counts for each mutation type were used for signature extraction using sigfit. A range of signatures (1-7) was given for possible signature extraction. Sigfit highlights the best number of signatures (as shown in red; signatures = 2), based on a cosine similarity metric that compares the original catalog of mutational signatures and the sigfit inferred signatures, as previously described in [43].

bioRxiv preprint doi: https://doi.org/10.1101/2023.03.06.531392; this version posted March 8, 2023. The copyright holder for this preprint (which was not certified by peer review) is the author/funder, who has granted bioRxiv a license to display the preprint in perpetuity. It is made available under aCC-BY 4.0 International license.

Fig S6

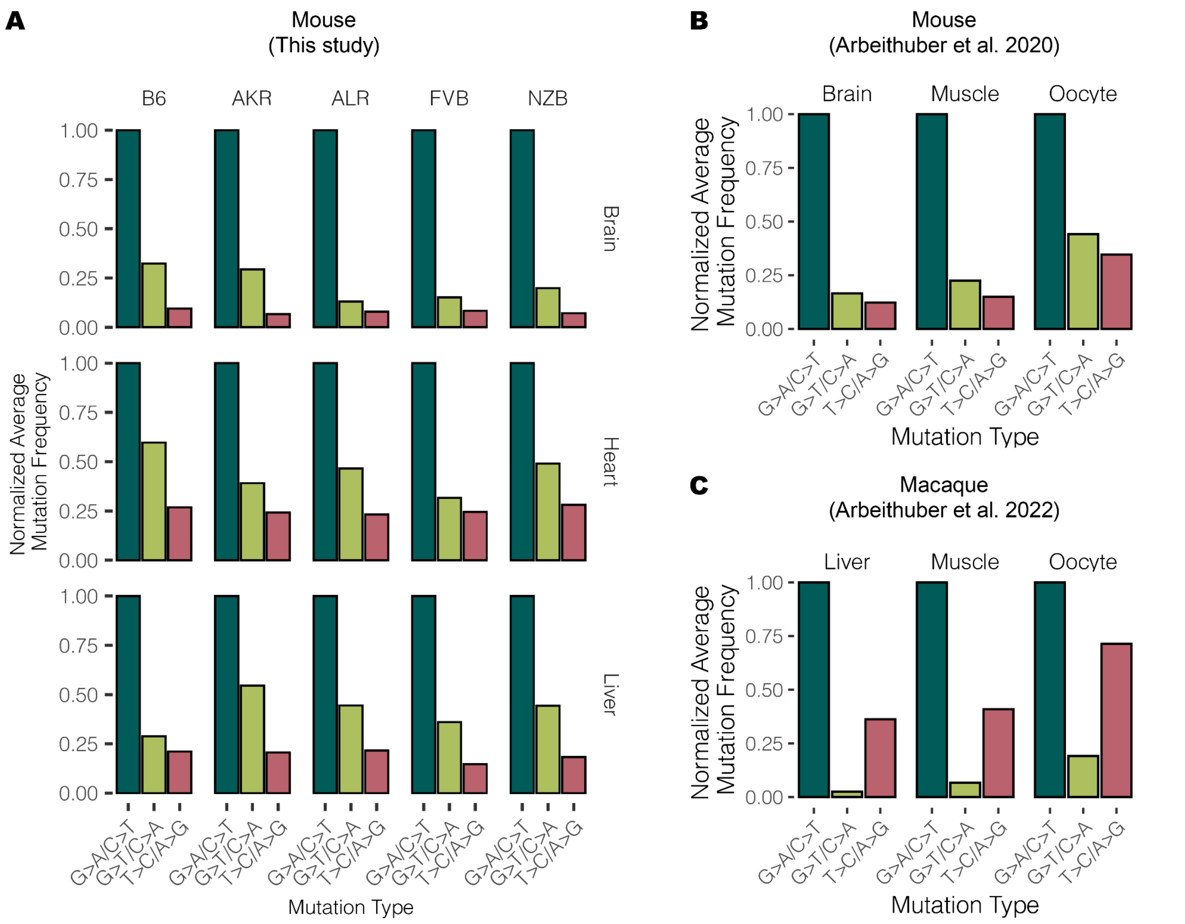

Fig S6: Mutation frequencies for macaque and mouse tissues in aged mice. The average frequency for point mutations was calculated as the total count of mutations of the given type divided by the duplex bp depth for the reference nucleotide. These average mutation frequencies were then normalized to the G>A/C>T mutation type, which is consistently the most abundant. Mutations with a frequency > $1x10^{-3}$ were excluded from this analysis. (A) The normalized average mutation frequency for the three most abundant mutations in this study across all experimental conditions. (B) The normalized average mutation frequency across three mouse tissues in Arbeithuber et al. 2020[32]. (C) The normalized average mutation frequency across macaque tissues in Arbeithuber el al. 2022 [33].

bioRxiv preprint doi: https://doi.org/10.1101/2023.03.06.531392; this version posted March 8, 2023. The copyright holder for this preprint (which was not certified by peer review) is the author/funder, who has granted bioRxiv a license to display the preprint in perpetuity. It is made available under aCC-BY 4.0 International license.

Fig S7

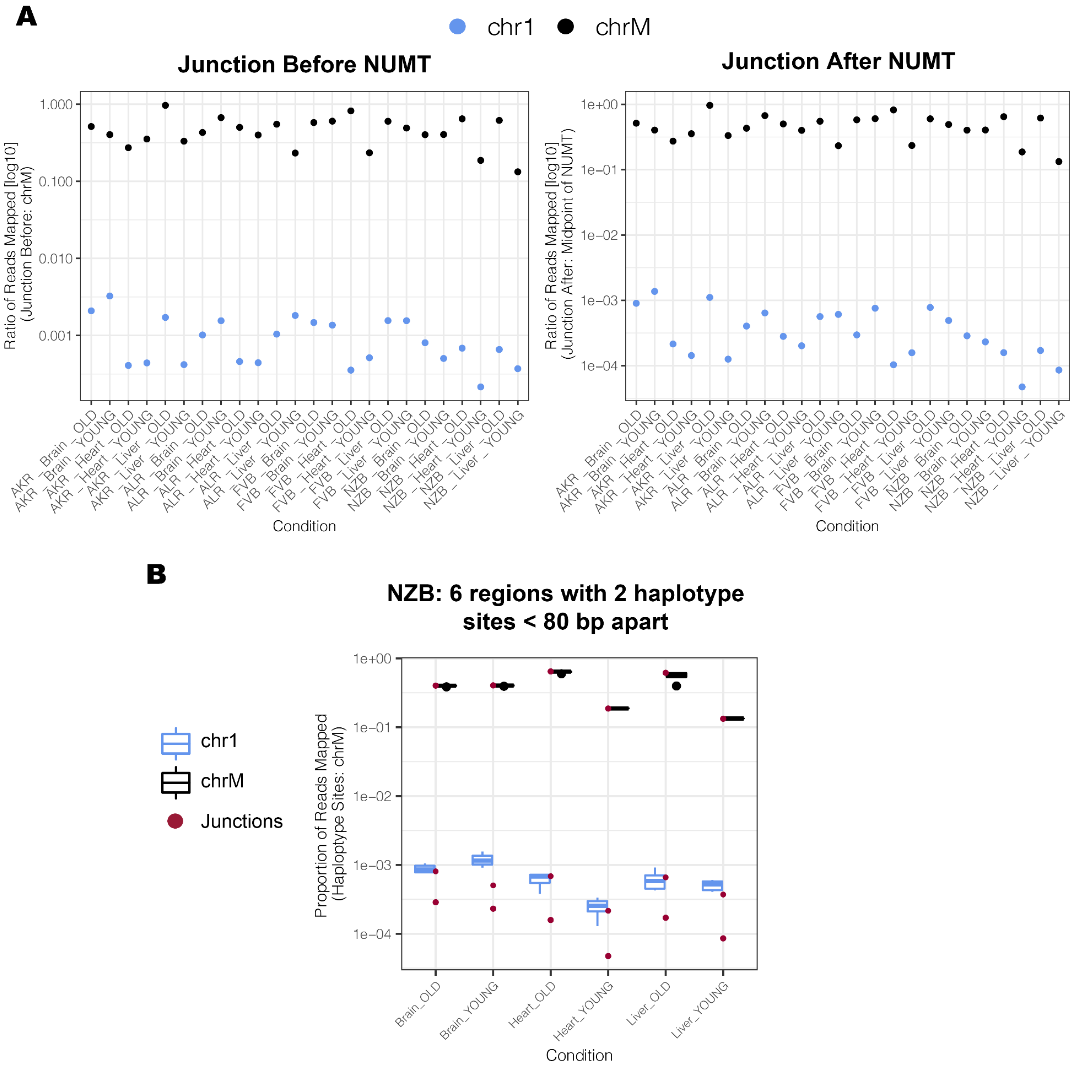

Fig S7: Estimation of NUMT contamination. (A) To estimate for NUMT contamination paired end duplex sequencing reads were remapped to mm10 with an unmasked chr1 NUMT. The number of reads that map to 10 bp upstream and 10 bp downstream of the NUMT region, referred to as the junction regions, was calculated. The figures denote the number of duplex reads that map to the junction regions divided by the average duplex depth of the corresponding region in the mt-genome with the chr1 NUMT masked (blue). Shown is also the number of reads that map to the corresponding junction regions in the mt-genome divided by the average duplex depth of the corresponding region in the mt-genome with the chr1 NUMT masked (black). (B) Duplex sequencing reads for the NZB strain were remapped using a NZB reference mt-genome. Six clusters of haplotype sites (regions where more than one haplotype site exists in a read) were identified. The distribution of the proportion of reads mapping the chr1 and those mapping to the mt-genome are depicted in boxplots. The proportion of reads mapping to chr1 and the mt-genome at the junction sites (shown in A) are represented in red.
